# Availability of a Flavored Beverage and Impact on Children’s Hydration Status, Sleep, and Mood

**DOI:** 10.3390/nu13061757

**Published:** 2021-05-21

**Authors:** Michael R. Szymanski, Gabrielle E. W. Giersch, Margaret C. Morrissey, Courteney L. Benjamin, Yasuki Sekiguchi, Ciara N. Manning, Rebecca L. Stearns, Douglas J. Casa

**Affiliations:** 1 Department of Kinesiology, Korey Stringer Institute, University of Connecticut, Storrs, CT 06269, USA; gabrielle.giersch@uconn.edu (G.E.W.G.); margaret.morrissey@uconn.edu (M.C.M.); cbenjami@samford.edu (C.L.B.); yasuki.sekiguchi@uconn.edu (Y.S.); ciara.manning@uconn.edu (C.N.M.); rebecca.stearns@uconn.edu (R.L.S.); 2 Department of Kinesiology, Samford University, Birmingham, AL 35229, USA

**Keywords:** euhydration, children, urine, thirst

## Abstract

Euhydration remains a challenge in children due to lack of access and unpalatability of water and to other reasons. The purpose of this study was to determine if the availability/access to a beverage (Creative Roots^®^) influences hydration in children and, therefore, sleep quality and mood. Using a crossover investigation, 46 participants were randomly assigned to a control group (CON) or an intervention group and received Creative Roots^®^ (INT) for two-week periods. We recorded daily first morning and afternoon urine color (Ucol), thirst perception, and bodyweight of the two groups. Participants reported to the lab once per week and provided first morning urine samples to assess Ucol, urine specific gravity (USG), and urine osmolality (Uosmo). Participants also completed the questionnaires Profile of Mood States-Adolescents (POMS-a) and Pittsburgh Sleep Quality Index (PSQI). Dependent *t*-tests were used to assess the effects of the intervention on hydration, mood, and sleep quality. Uosmo was greater and Ucol was darker in the control group (mean ± SD) [Uosmo: INT = 828 ± 177 mOsm·kg^−1^, CON = 879 ± 184 mOsm·kg^−1^, (*p* = 0.037], [Ucol:INT = 5 ± 1, CON = 5 ± 1, *p* = 0.024]. USG, POMS-a, and PSQI were not significant between the groups. At-home daily afternoon Ucol was darker in the control group [INT = 3 ± 1, CON = 3 ± 1, *p* = 0.022]. Access to Creative Roots^®^ provides a small, potentially meaningful hydration benefit in children. However, children still demonstrated consistent mild dehydration based on Uosmo, despite consuming the beverage.

## 1. Introduction

There is an increasing body of evidence showing that children are inadequately hydrated [1]. A recent review examining water intake across 19 countries found that about 60% of children aged 4–13 years did not meet water intake guidelines [2]. Specifically, in the United States, Kenny et al. [3] examined the prevalence of insufficient hydration using urine osmolality and found that over 50% of their sample (*n* = 4134) aged 6–19 years were inadequately hydrated. There are many barriers that may influence the consumption of water among children including, but not limited to, lack of drinking water accessibility while at school, unpalatable water, and the availability of competitive beverages (e.g., sugar-sweetened beverages, 100% fruit and vegetable juices) [4].

The hydration status has been associated with mood state, as well as sleep quality [5,6,7]. Evidence has demonstrated that dehydration adversely affects mood, with increases in tension and anxiety, as well as fatigue [5,6]. Additionally, Fadda et al. [6] found a negative correlation between hydration status (assessed as urine osmolality) and vigor, indicating that hydration is beneficial in improving vigor. A short sleep duration has also been linked to a poor hydration status, and those with a sleep duration of 6 h were presented higher urine specific gravity and greater odds of being dehydrated [7]. However, the aforementioned study [7] examined adult male participants. There is limited evidence on the hydration status and sleep quality in children.

Water is an essential component for many physiological functions within the human body and plays a critical role in thermoregulation, cardiovascular function, as well as transportation of nutrients and waste production [8]. Inadequate hydration can lead to numerous negative health effects [3,8]. Even mild to moderate dehydration has been associated with sleepiness, headaches, and muscle weakness [8]. Severe dehydration has been associated with irritability and sleepiness, especially in children and infants, low blood pressure, and rapid heartbeat [8]. These negative health effects can be detrimental to the development of children and warrant further access to water, specifically during school hours.

In an attempt to combat the hydration barriers in children, the Kraft Heinz Company (Chicago, IL, USA) developed a coconut water-based beverage. Therefore, the purpose of the present study was to determine if the accessibility to the beverage influences hydration, as well as sleep quality and mood status, in children. We hypothesized that having access to this beverage would improve the hydration status and, therefore, overall mood and sleep quality.

## 2. Materials and Methods

### 2.1. Beverage

The Kraft Heinz Company (Chicago, IL, USA) developed an 8.5 oz (251 mL) coconut water-based beverage (15% juice) with one gram of sugar and 11 calories, called Creative Roots^®^.

#### Experimental Approach to the Problem

We utilized a randomized crossover study design to guide data collection. All procedures were reviewed and approved by University of Connecticut’s Institutional Review Board (H19-212). The participants provided verbal assent, and their parents or guardians provided written consent prior to participation.

### 2.2. Participants

A sample of 46 children (*n* = 23 males, *n* = 23 females) ranging from 7 to 12 years of age (10 ± 2 years old) at the time of consent volunteered for this study. Participants were excluded from the study if they had a history of chronic kidney disease, diabetes, sleep disorder, if used medication that might affect water balance (e.g., diuretics, laxatives, antacids, antihistamines, NSAIDs, blood pressure medication) or mood and anxiety (e.g., antidepressants, anxiolytics, beta-blockers, ADHD medication), as well as other medications that might cause urine color changes (e.g., isoniazid, sulfasalazine, metronidazole, nitrofurantoin, amitriptyline, cimetidine, indomethacin, zaleplon, methocarbamol, metoclopramide, warfarin, rifampin, and phenazopyridine). Participants were also excluded from the study if they did not find any of the drinks palatable.

### 2.3. Overview of the Study Procedures

Participants reported to the lab once a week over 7 weeks for a total of 7 lab visits. A timeline and overview of the study procedures can be seen in Table 1. Participants began the study with a familiarization visit in which they completed all laboratory procedures and were given detailed instructions by a research team member about how to properly complete data collection at home. The participants returned to the lab at least one week later and, following laboratory procedures, were randomly assigned to either an intervention group or a control group. The intervention group received 40 bottles of Creative Roots© (Kraft Heinz Company, Chicago, IL, USA). The parents/guardians of the participants were instructed to ensure that the participants had the option to drink the beverage at each meal (i.e., breakfast, lunch, and dinner), as well as to allow the participants to drink the beverage ad libitum throughout the day. Participants in the intervention group returned their empty and unused bottles and were given a new batch of 40 bottles each week. The control group did not receive the beverages and were not given any further instructions about fluid or dietary intake.

Following this two-week period, both groups (i.e., intervention and control groups) entered a washout week, in which no beverages were given, and participants were instructed to cease the at-home data collection. Following the washout week, participants returned to the lab and were assigned to their new groups (i.e., the intervention group became the control group and vice versa). Both groups were instructed to resume their bi-daily at-home assessments.

### 2.4. Weekly Lab Visits

Participants reported to the lab once a week over seven weeks for a total of seven lab visits. On the first lab visit, the participants were familiarized with the testing variables and taught how to assess their hydration. First, participants were given an iPod touch (Apple Inc., Cupertino, CA, USA) with the Qualtrics Survey Application (Qualtrics, Provo, UT, USA) to self-assess their daily morning and afternoon urine color, thirst perception, and bodyweight throughout the duration of the study. Additionally, participants tasted the Creative Roots^®^ drink to ensure palatability. If the participant did not like the drink, they were excluded from the study; however, all participants enjoyed the beverage. Weekly lab visits involved a hydration assessment, the completion of the Profile of Mood States adolescent (POMS-a) [9] and of the Pittsburg Sleep Quality Index (PSQI) [10] questionnaires. For the hydration assessments, participants were provided with a urine collection cup, instructed to collect their first morning urine, and bring the sample to the laboratory. Urine color was assessed with the urine color chart that has been validated for children [11]. Urine specific gravity (USG) was assessed using a handheld refractometer (Reichert TS 400, Reichert Inc., Dewpew, NY, USA), and urine osmolality (Uosmo) was assessed using freeze-point depression (OsmoPRO^®^ Multi-Sample Micro-Osmometer; Advanced Instruments, Norwood, MA, USA). Thirst perception was measured using a previously validated [12,13] nine-point (1–9) Likert scale, where 1 is “Not Thirsty at All” and 9 is “Very, Very Thirsty”. Additionally, body mass was assessed at this time (Defender R7000 Xtreme; OHAUS Corp., Parsippany, NJ, USA). In order to limit parent and peer influence during the POMS-a and the PSQI, the participants were instructed to direct all questions to a member of the research team.

### 2.5. At Home Data Collection

Participants were given an iPod touch (Apple Inc., Cupertino, CA, USA) with the Qualtrics Survey Application (Qualtrics, Provo, UT, USA) to self-assess their daily morning and afternoon urine color, thirst perception, and bodyweight throughout the duration of the study. Following urination in the urine collection cup at home, urine color was assessed by the participant with the urine color chart in paper format or as a picture on the iPod. Bodyweight was measured at home with a bodyweight scale (BalanceFrom LLC., Los Angeles, CA, USA), and the perceived level of thirst was assessed immediately upon waking in the morning and upon return home from school in the afternoon (i.e., between 1600 and 1959 h) [14].

### 2.6. Statistical Analysis

Our sample size calculation was based on the comparison of a low water intake intervention vs. an ad libitum water intake in preadolescent children [1]. To establish an estimate of power and to project a proper sample size, the values for differences in 24 h urine osmolality from a low water intake intervention and an ad libitum water intake were utilized. It was determined that urine osmolality values for low water intake were 912 ± 199 mOsm·kg^−1^, and those for an ad libitum water intake were 790 ± 257 mOsm·kg^−1^. For a matched-pairs test with 0.05 alpha level, effect size of 0.52, and desired power level of 0.95, the estimated sample size would be a minimum of 42 participants. We utilized the power calculation software G*power 3.1 to calculate the required sample size needed for this study.

Data are reported as means and standard deviations (M ± SD). Dependent *t*-tests were used to determine differences between the intervention (INT) and the control (CON) groups for urine color, Uosmo, USG, thirst sensation, PSQI, POMS-a, and daily percent bodyweight changes [(Afternoon bodyweight–morning bodyweight)/morning bodyweight (×100)]. Alpha level was set a priori at 0.05. All statistical analyses were computed using SPSS (SPSS Statistics version 25, IBM Corp., Armonk, NY, USA).

## 3. Results

**Figure 1:**
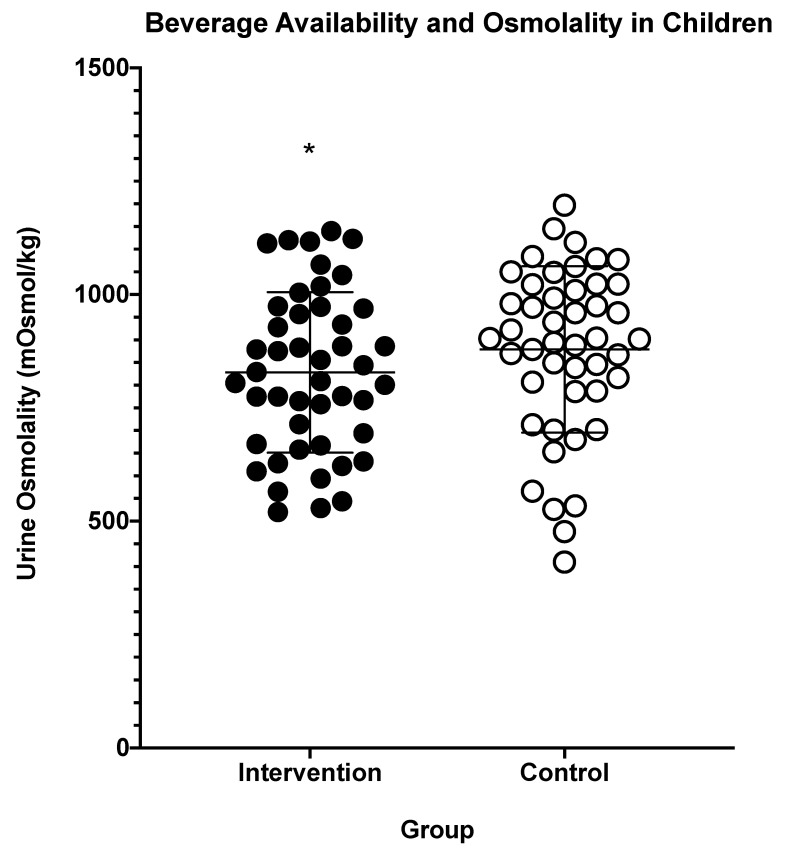
Values are averages of lab visit urine osmolality in the intervention and control groups; *n* = 46 after a two-week period. Intervention = flavored beverage. * indicates statistical significance (*p* < 0.05). The lines within the individual data points represent each group’s mean and standard deviation.

**Figure 2:**
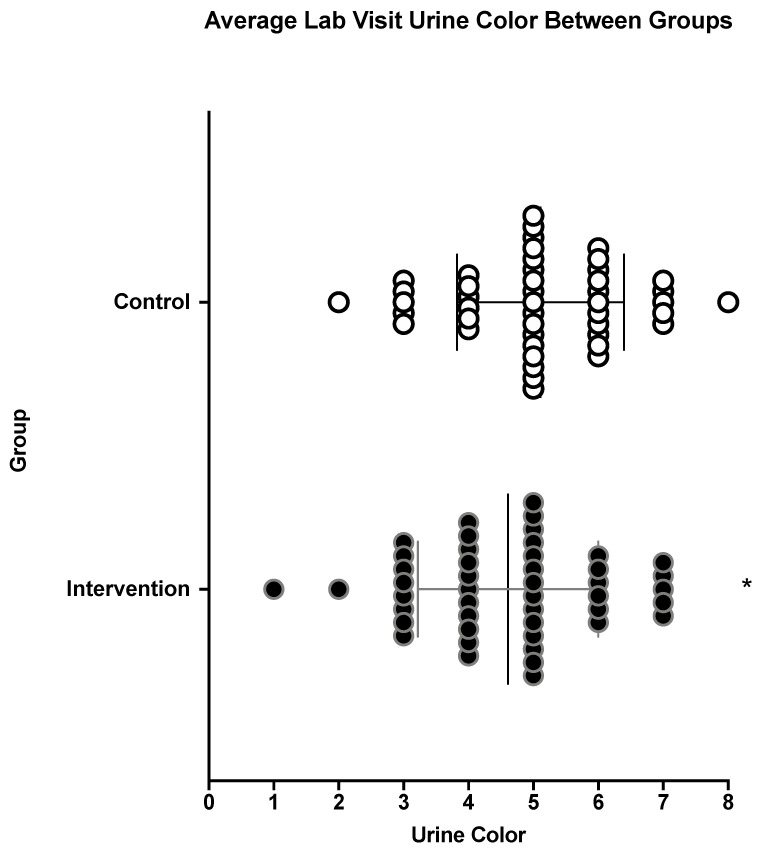
Values are averages of lab visit urine color for the intervention and the control groups; *n* = 46 after a two-week period. Intervention = flavored beverage. * indicates statistical significance (*p* < 0.05). The lines within the individual data points represent each group’s mean and standard deviation.

**Figure 3:**
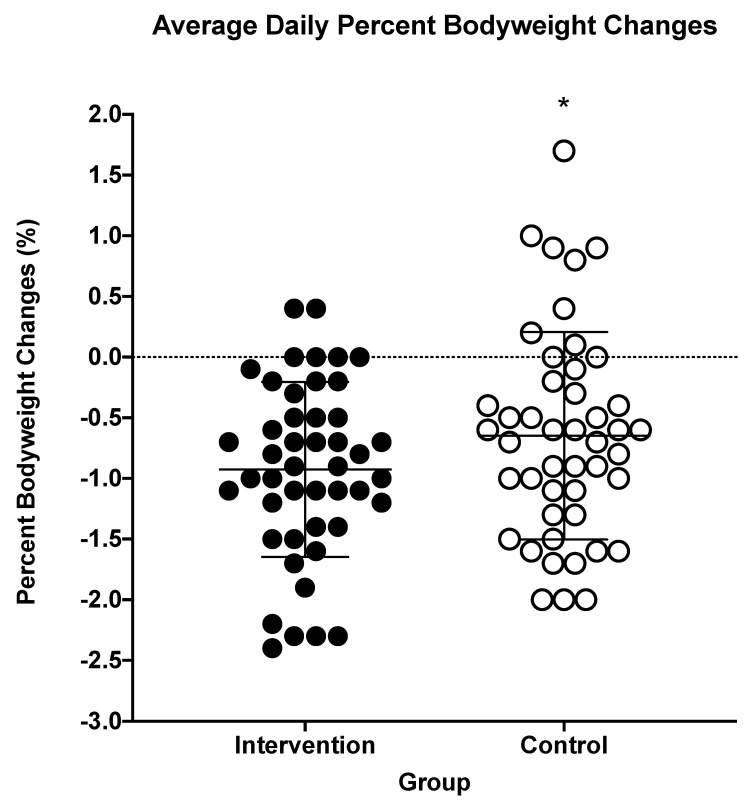
Values are average daily bodyweight percent changes in the intervention and in the control groups; *n* = 46. * indicates statistical significance (*p* < 0.05). The lines within the individual data points represent each group’s mean and standard deviation.

INT samples presented lower Uosmo and lighter urine color compared to CON samples, as shown in Figure 1 and Figure 2, respectively. The afternoon urine color from at-home measures was lighter for the INT group compared to the CON group, as shown in Table 2. INT also had greater percent body mass loss, as shown in Figure 3. For all other measures taken at home, there were no differences, including for morning urine color as well as morning and afternoon thirst sensation, as shown in Table 2. INT did not show any effect on USG, thirst perception, POMS-a total score, or PSQI [USG: INT = 1.023 ± 0.005, CON = 1.024 ± 0.005, *p* = 0.091], [thirst: INT = 4 ± 1.6, CON = 4 ± 1.6, *p* = 0.657], [POMS-a: INT = −1.04 ± 5.6, CON = −0.26 ± 6.6, *p* = 0.233], [PSQI: INT = 2.91 ± 1.8, CON = 3.07 ± 1.5, *p* = 0.425].

## 4. Discussion

These results suggest that the INT group was able to improve their hydration status based on the decreased Uosmo and urine color from weekly laboratory visits. Although there was an observed decrease of dehydration in the INT group, both groups had clinical signs of dehydration from the spot samples provided (Uosmo > 800mOsm·kg^−1^). This would suggest that although the INT groups did see improvements to their Uosmo, the INT condition was not sufficient to attain clinical euhydration. Despite being different at a statistically significant level between groups, urine color during the lab visits was similar for both groups, having a value of ~5 and a mean difference of only 0.5. Furthermore, USG and thirst perception during the lab visits were not significantly different between groups, suggesting that having accessibility to the Creative Roots^®^ beverage may only improve Uosmo. However, the improvements in Uosmo may suggest that the Creative Roots^®^ beverage offers a potential benefit for hydration, due to the validity of Uosmo for assessing hydration [15]. The at-home morning urine color and morning and afternoon thirst perception did not show any change in the INT group when the participants were self-assessing their thirst perception and urine color.

Our results are similar to those of Khan et al. [1] who examined hydration markers during a 4-day water intake intervention in children with a prescribed low intake, high intake, and ad libitum intake of fluid. The authors reported Uosmo and urine color values in their ad libitum group that would approach the threshold for clinical dehydration, similar to what we observed in our participants, who were able to consume fluid ad libitum [1]. This similarity may suggest that children with an ad libitum beverage intake may not see improvements in hydration and that a prescribed beverage intake may be more beneficial for improving hydration.

Interestingly, the at-home data (i.e., morning and afternoon urine color, bodyweight, and thirst perception) showed contradictory results. First, the afternoon urine color was lighter for the INT group; however, due to the low reported mean difference, we speculate that it is unlikely that these results have any clinical significance. Additionally, daily percent bodyweight changes were found to be statistically significant between the groups. The intervention group presented greater differences in daily percent bodyweight changes when compared to the control group, suggesting the intervention group was less hydrated than the control group. Bodyweight changes have been noted as one of the more practical markers for hydration, whereas day-to-day bodyweight losses of more than 1% can be an indicator of dehydration [16]. Although the intervention group did not lose over 1% of bodyweight, the group still lost more bodyweight than the control group, which is contradictory with their afternoon urine color measures. However, the bodyweight changes may be related to a reduced consumption of sugary beverages, thus a reduced caloric intake, due to having access to Creative Roots^®^. Prior research has demonstrated that reducing sugary beverage intake and replacing these beverages with non-caloric ones can result in reduced weight gain [17,18].

Contrary to previous research, we did not find any differences in mood between INT and CON, using the POMS-a. Fadda et al. [6] found that serving children 300–500 mL of water during the school day can improve their mood. This was a prescribed amount, whereas in the present study we utilized ad libitum fluid consumption and only assessed mood in children once per week, which may explain why we did not find any differences between our groups. This may suggest that acute changes in hydration may impact mood, but it is possible that those differences are not observable when only assessed one time per week. We also did not observe any differences in sleep quality, using the PSQI, between the intervention and the control groups despite the intervention group having a lighter urine color in the afternoon. This finding may simply be due to the intervention not changing the hydration status. Further research is warranted, as there is little evidence available regarding hydration status and sleep quality in children.

## 5. Limitations

There are several limitations to the present study. First, the intervention group was not prescribed a specific amount of fluid. We required the participants to have drinks available and accessible at each meal (i.e., breakfast, lunch, and dinner), as well as ad libitum throughout the day, but did not require them to consume specific amounts. Second, we did not ask the children or their parents to record what was consumed throughout each week. We also used first morning urine samples for hydration analysis (i.e., urine color, USG, Uosmo) in all lab visits in order to reduce scheduling conflicts with the participants and their caretakers. Recent literature suggests that Uosmo of afternoon urine samples is more representative of values observed in 24 h urine collection [14]. Lastly, the daily log data collection was not supervised by the research staff, though research participants were provided detailed instructions to promote compliance.

## 6. Conclusions

Our results indicate that having access to Creative Roots^®^ seems to produce a small but potentially meaningful benefit in hydration, as indicated by Uosmo and urine color. However, even with the observed improvements, children were still consistently mildly dehydrated regardless of the group. Our data show the intervention group did improve some biomarkers of hydration, but no effect was observed in at-home measures, mood, or sleep. Further research is warranted using a prescribed amount of fluid to determine if the beverage improves the hydration status and monitoring dietary and fluid intake during the entirety of the study.

## Figures and Tables

**Table 1:** Dependent variables and collection time points.

Variable	Familiarization	Baseline	Daily Data Collection During Weeks 1, 2, 4, 5 (at Home)	Weekly Visit with Researchers (End of Weeks 1–5)
Body mass	X	X	X	X
Urine Sample	X			X
Urine Specific Gravity	X			X
Urine Osmolality	X			X
AM Urine Color		X	X	X
PM Urine Color		X	X	X
Thirst Sensation	X	X	X	X
Sleep Questionnaire	X	X		X
Mood (POMS)	X	X		X

**Table 2:** At-home daily log of morning and afternoon urine color and morning and afternoon thirst perception.

	Morning Urine Color	Afternoon Urine Color	Morning Thirst Perception	Afternoon Thirst Perception
Intervention	4 ± 1	3 ± 1 *	4 ± 1	3 ± 1
Control	4 ± 1	3 ± 1	4 ± 1	3 ± 1

Intervention = flavored beverage. * indicates statistical significance (*p* < 0.05). *n* = 46

## Data Availability

Data available only on request due to ethical restrictions.
